# Machine-Learning Approach for Identifying Arsenic-Contamination Hot Spots: The Search for the Needle in the Haystack

**DOI:** 10.1021/acsestwater.4c00422

**Published:** 2024-07-15

**Authors:** Marinus E. Donselaar, Sufia Khanam, Ashok K. Ghosh, Cynthia Corroto, Devanita Ghosh

**Affiliations:** † Department of Geoscience and Engineering, Delft University of Technology, 2628 CN Delft, The Netherlands; ‡ Environment and Population Research Center (EPRC), Mohakhali, Dhaka 1000, Bangladesh; § Mahavir Cancer Sansthan and Research Centre, Patna 801505, India; ∥ Centro de Estudios Transdisciplinarios del Agua (CETA), Universidad de Buenos Aires, C1053ABH Buenos Aires, Argentina; ⊥ Department of Water Management, Delft University of Technology, 2628 CN Delft, The Netherlands

In the 40 years since the relation between arsenic (As) toxicity and groundwater extraction was first documented from the Holocene alluvial basin of West Bengal, India,^1^ we have become more aware that groundwater contamination with naturally occurring (geogenic) As poses a serious health threat of global proportions.^2^ With the aim of implementing effective and sustainable mitigation strategies, research into the occurrence and location of toxic As levels in drinking and irrigation water and in the food chain provided insight into all aspects of the As-contamination issue, including (a) geogenic As provenance in volcanic and metamorphic rocks, hydrothermal additions to groundwater and hot springs, and weathering of rocks in orogenic mountain belts, (b) its accumulation in sedimentary-basin aquifers, (c) the mobilization and transport of the contaminant into the groundwater, and (d) the associated health risks of sustained As ingestion for >200 million people around the world.^3,4^ A wide range of potential As-mitigation measures have been proposed over the years, ranging from in situ chemical and biological oxidative processes for immobilizing As to subsequent filtration methods and social awareness programs for the affected population.^5−7^

The apparently random spatial variation of groundwater As concentrations in alluvial basins underpins the enigmatic nature of the As hot spot occurrence as the large remaining challenge that hampers the focused and economically viable application of sustainable mitigation measures. It is comparable to a well-equipped fire brigade at a loss to extinguish the raging fire, unaware of the exact coordinates of the peril. In terms of the surface area and number of people in potential harm, Holocene alluvial basins such as the Ganges-Brahmaputra Basin in southeast Asia with a combined drainage area of 1.6 × 10^6^ km^2^ are by far the largest As-contamination-prone environment. To date, attempts to locate sites with high levels of As contamination in groundwater in the vast area of alluvial basins focused on contour mapping based on geostatistical interpolation of As-concentration spot measurements from tube wells. These maps offer a global but unfocused view of high As concentrations at best and, depending on the interpolation algorithm (Kriging, inverse-distance weighting), erroneously feature apparent As peaks in ridges or in so-called “bull’s eye” patterns around data points.^8,9^ A promising new research approach is the construction of predictive As-distribution maps with random forest geospatial machine-learning algorithms that incorporate a wide variety of soil types as predictor variables and result in smoother maps that cover large areas of potential As risk.^10,11^

In this Viewpoint, we outline the path toward efficient As hot spot mapping with the aid of machine-learning techniques that take into account the pivotal, interacting factors that control the release and accumulation of As in sedimentologically confined units: (a) alluvial geomorphology that comprises the heterogeneity between geomorphological units and the inherent porosity–permeability anisotropy that controls groundwater flow paths and recharge efficiency and (b) biogeochemical processes that favor the release of As from its solid state and subsequent entrapment in isolated porous geomorphological units in the anisotropic aquifer domain. The approach is analogous to the exploration of hydrocarbon accumulations in porous and permeable sediment bodies by reservoir modeling of the source rock–reservoir rock–cap rock triad.

**Figure 1:**
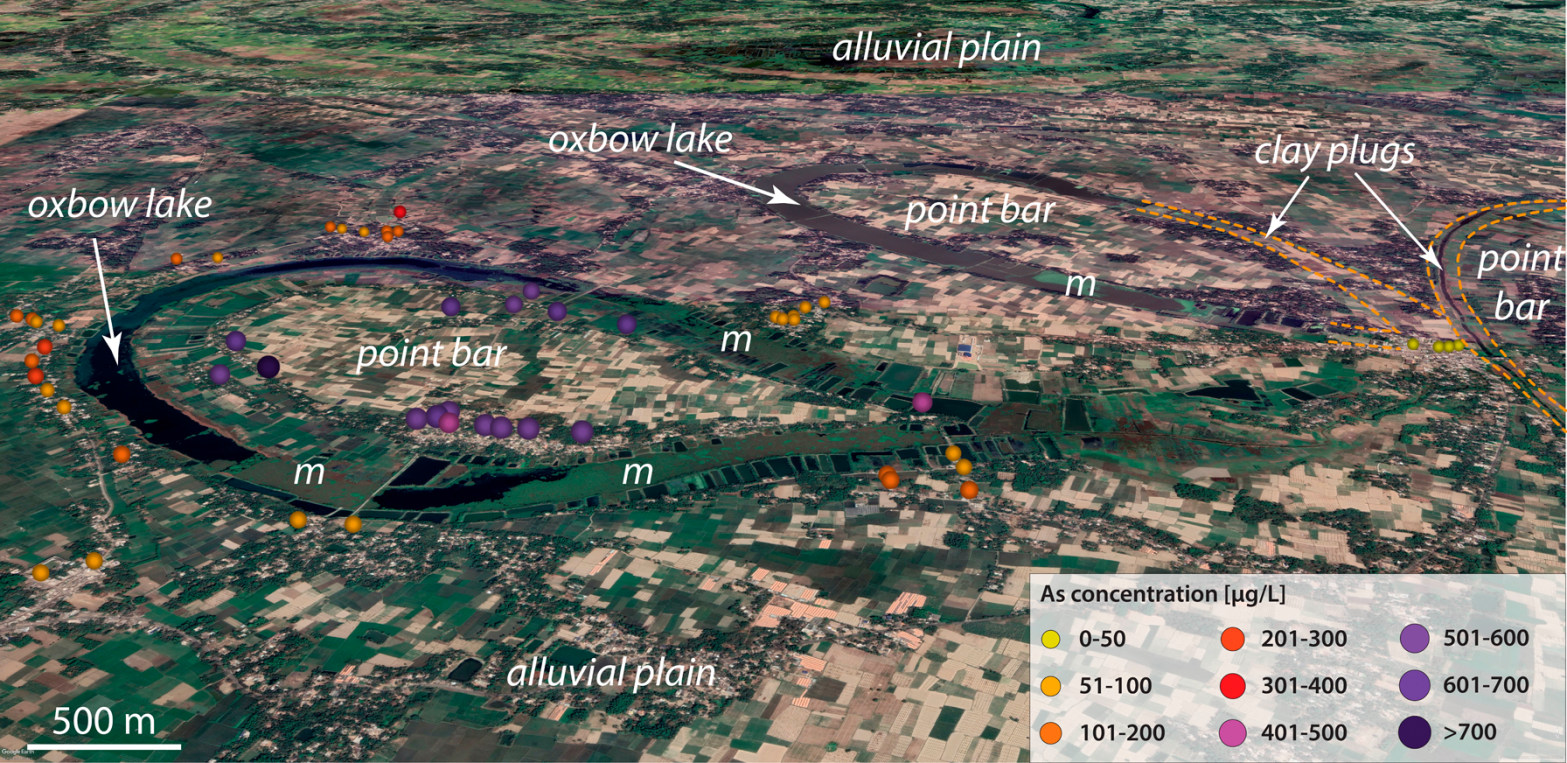
Alluvial geomorphology with teardrop-shaped sandy point bars (each with a surface area of ∼2 km^2^) encompassed by abandoned meandering-river bends (oxbow lakes) and (partly) sediment-filled counterparts (clay plugs). *m* indicates invasive macrophytes (*Eichhornia crassipes* sp. and *Hydrilla verticillata* sp.) on the oxbow-lake surface. Point bars 7 m above the surrounding alluvial plain. Population nuclei (average population density of 1093 km^–2^) on elevated point bars and sandy outer river banks. As-concentration data from shallow (≤40 m deep) tube wells. Highest As concentrations in an enclosed point-bar aquifer.^13^ Jamuna River Basin, West Bengal, India. 22°58′43.58″N, 88°38′3.31″E. Map Data: Google, © 2024 Maxar Technologies. Image date: February 28, 2021.

Recent research advances indicate that detached, abandoned meandering-river bends (or oxbow lakes), their fine-grained sediment-filled counterparts (or clay plugs), and associated sand-prone point bars are potential sites with high levels of As contamination in the alluvial-basin landscape on a global scale (Figure 1).^12,13^ Porous and permeable sandy point bars stand out, induced by differential compaction, as topographical high grounds in the alluvial landscape, whereas fine-grained alluvial plain and clay-plug sediment is compacted, thereby reducing its porosity and permeability. Population nuclei on elevated point bars provide protection from yearly monsoonal river inundation. Here, excess tube well groundwater extraction leads to pressure gradients and draw-up of As-contaminated water to the well heads.

The oxbow lake’s oxygen-deprived lower part of the water column (hypolimnion) stores organic carbon from dead biomass of invasive macrophytes eradicated by annual monsoon floods. This adds high-molecular weight dissolved organic carbon (HMW-DOC) to the oxbow-lake sediment. A high HMW:TOC ratio and a low total organic carbon (TOC) indicate microbial activity. Fecal markers suggest anthropogenic enrichment, promoting methane-producing microbes. The HMW-DOC reaches the oxygen-depleted aquifers and triggers the reduction of As(V) to As(III) and its release. Dissolved As(III) then migrates to sandy point bars by diffusion and advection along the porosity–permeability gradient, driven by gravity and clay compaction.^13,14^ The compacted alluvial plain and clay plug are the low-permeable envelope that forms a four-way closure around the point-bar reservoir, initially at the surface in the alluvial plain and, upon burial by continued fluvial sedimentation in the subsiding Holocene alluvial basin, also overlying the point-bar sand in the subsurface. The resultant anisotropic sedimentary architecture constrains the groundwater flow paths and strongly reduces the recharge efficiency in the aquifer domain of the enclosed pockets of porous point-bar sand, leading to the accumulation of As with concentrations on the order of 500 μg/L^13^ (Figure 1), i.e., far beyond the WHO-recommended maximum level of 10 μg/L. The point-bar/oxbow-lake/clay-plug geomorphological units are ubiquitous, with scattered locations in all major river channel belts in Holocene alluvial basins around the world, with a total areal extent of many millions of square kilometers.

**Figure 2:**
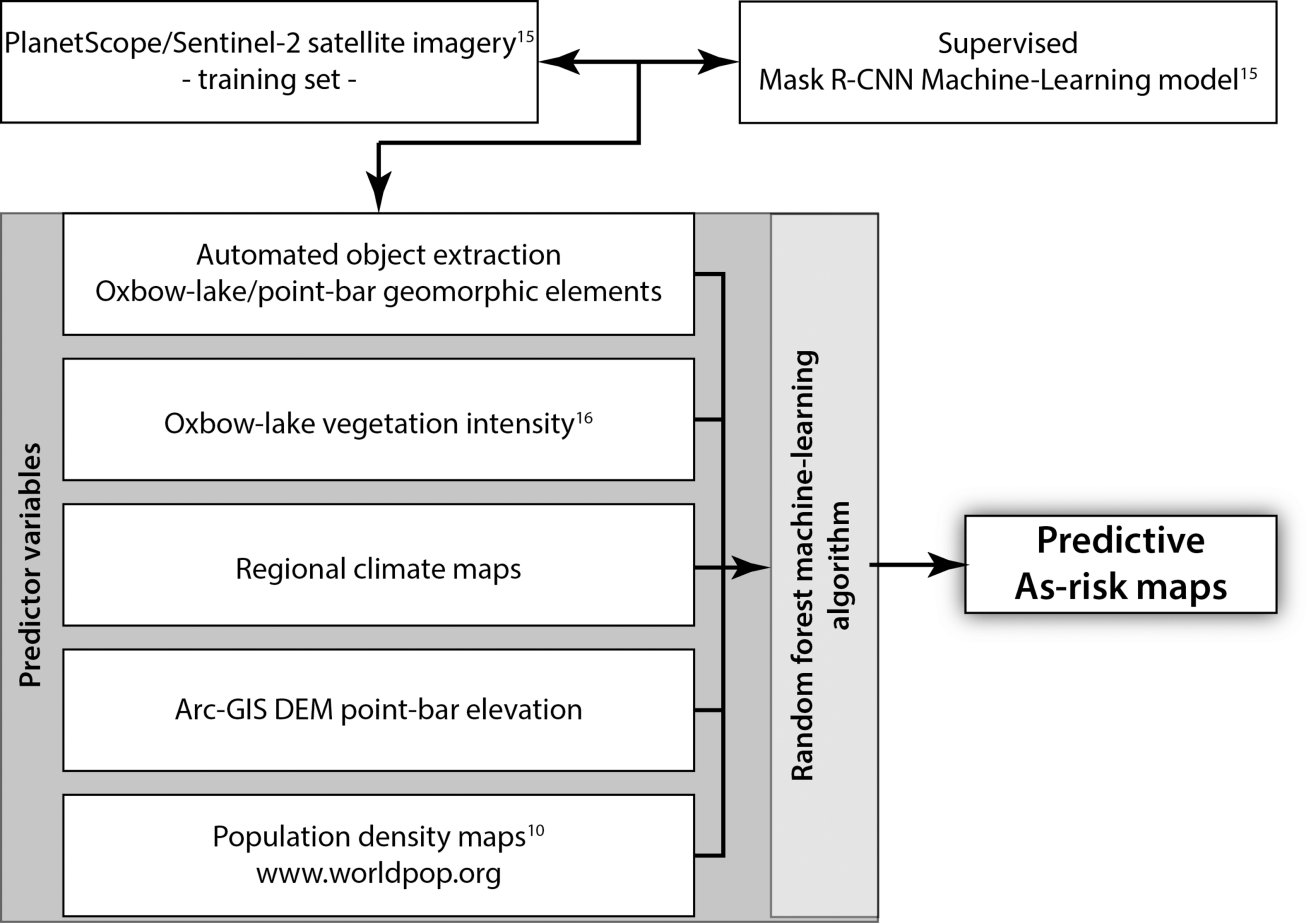
Machine-learning steps toward the automated production of As-risk maps.

With the knowledge that As-contamination hot spots are preferentially concentrated in porous and permeable point-bar sands, and with the remediation urgency for an efficient, rapid detection of similar geomorphological and associated contamination setting, the next step will be to apply a machine-learning technique for automatic As hot spot detection, i.e., finding the needle in the haystack, in the alluvial basins by a combination of (a) a mask region-based convolutional neural network (Mask R-CNN) model as a novel, state-of-the-art technique for the remotely sensed extraction and image segmentation of complex-shaped geomorphological objects such as point-bar/oxbow-lake units and (b) a Random Forest (RF) machine-learning classifier (Figure 2) with a set of predictor variables that narrow the myriad of geomorphological objects to those meeting the criteria for As hot spots. The supervised Mask R-CNN model, trained over Sentinel-2 or PlanetScope satellite imagery,^15^ has the ability to automatically produce detailed map views of similar geomorphological objects at alluvial-basin scale. Subsequently, the automatically generated map views are combined in a RF classifier (Figure 2) with a set of predictor variables meeting the criteria for As hot spot remediation targets: oxbow-lake vegetation intensity^16^ and climate setting for the estimation of the yearly addition of organic matter to the lake sediment, essential for the process of reductive dissolution of As,^14^ and ArcGIS-generated digital elevation models (DEMs) combined with population density maps^10^ in the potential hot spot areas to identify the coincidence of point-bar locations with topographic high grounds and population nuclei. The approach will yield predictive As-risk maps, which serve to pinpoint target areas for the focused application of mitigation measures. Available ground-truth As-concentration databases and biogeochemical and sedimentological information will serve as machine-learning training sets for the verification of high As concentrations in the predictive risk maps. To facilitate the rapid deployment and analysis of verification databases, which are at present dispersed among government agencies, local authorities, NGOs, and research institutes, we here advocate the centralized storage in freely accessible and searchable online databases, managed by data custodians such as the Central Ground Water Board (CGWB) in India and the Department of Public Health Engineering (DPHE) in Bangladesh.

Point-bar thicknesses in the alluvial plains are in the range of 8–12 m;^12^ hence, the proposed machine-learning methodology is limited to capturing the spatial distribution of As hot spots in the uppermost part of the Holocene stratigraphy. However, in the course of fluvial sedimentation in the subsiding alluvial basin, meandering-river sediment accumulation creates a thick Holocene fluvial stratigraphy (on the order of 100 m in the Ganges Brahmaputra alluvial basin^18^) with high potential of sand-on-sand vertical connectivity of point-bar deposits^12,19^ and, hence, shallow tube wells with a depth of ∼30 m are very likely to tap from deeper-lying point-bar sands.^12^

The proposed machine-learning approach has a limited number of dedicated predictor variables based on the principle of As accumulation in geomorphologically well-defined objects, which is much more manageable than the extensive number (≤17) of soil type variables used to date^10,11^ without relation to geomorphological anisotropy. The approach is versatile in the sense that, if other geomorphological elements such as river banks or levees^20^ systematically prove to act as sinks for dissolved As, the workflow can be extended to capture these morphological elements. Finding the needle in the haystack will lead to a focused, localized application of groundwater treatment technology in As hot spots, thereby potentially saving lives, reducing operational costs, and limiting the environmental footprint.
